# Flow Field Perception of a Moving Carrier Based on an Artificial Lateral Line System

**DOI:** 10.3390/s20051512

**Published:** 2020-03-09

**Authors:** Guijie Liu, Huanhuan Hao, Tingting Yang, Shuikuan Liu, Mengmeng Wang, Atilla Incecik, Zhixiong Li

**Affiliations:** 1 Department of Mechanical and Electrical Engineering & Key Laboratory of Ocean Engineering of Shang Dong Province, Ocean University of China, Qingdao 266100, China; hhh@stu.ouc.edu.cn (H.H.); 17865324126@163.com (T.Y.); lsk0308@163.com (S.L.); wmm@stu.ouc.edu.cn (M.W.); 2 Department of Naval Architecture, Ocean and Marine Engineering, University of Strathclyde, Glasgow G1 1XQ, UK; atilla.incecik@strath.ac.uk; 3 School of Mechanical, Materials, Mechatronic and Biomedical Engineering, University of Wollongong, Wollongong, NSW 2522, Australia; zhixiong_li@uow.edu.au

**Keywords:** artificial lateral line system, fluid velocity, flow angle, carrier speed, neural network

## Abstract

At present, autonomous underwater vehicles (AUVs) cannot perceive local environments in complex marine environments, where fish can obtain hydrodynamic information about the surrounding environment through a lateral line. Inspired by this biological function, an artificial lateral line system (ALLS) was built on a moving bionic carrier using the pressure sensor in this paper. When the carrier operated with different speeds in the flow field, the pressure distribution characteristics surrounding the carrier were analyzed by numerical simulation, where the effect of the flow angle between the fluid velocity direction and the carrier navigation direction was considered. The flume experiment was carried out in accordance with the simulation conditions, and the analysis results of the experiment were consistent with those in the simulation. The relationship between pressure and fluid velocity was established by a fitting method. Subsequently, the pressure difference method was investigated to establish a relationship model between the pressure difference on both sides of the carrier and the flow angle. Finally, a back propagation neural network model was used to predict the fluid velocity, flow angle, and carrier speed successfully in the unknown fluid environment. The local fluid environment perception by moving carrier carrying ALLS was studied which may promote the engineering application of the artificial lateral line in the local perception, positioning, and navigation on AUVs.

## 1. Introduction

Owing to the complex nature of marine environments, humans have always been developing marine equipment and precision instruments with various functions in order to exploit and explore ocean resources. Autonomous underwater vehicles (AUVs) not only need to maintain stability in navigation but also to conduct environment perception and navigation positioning. Currently, optical sensors, acoustic systems, and global positioning system (GPS) has been used for AUVs to enable their operation in underwater environments; however, due to the fact of their limitations, such as environments which limit the use of GPS, it is difficult to effectively perform environment perception and navigation positioning.

In a complex and ever-changing marine environment, fish can sense the fluid environment through lateral lines to hunt and avoid enemies. Biologists have discovered that all fishes and some aquatic amphibians have a special sensory-mechanical sideline that can detect and process tiny water movements, enabling the perception of local environments. The basic sensory unit of the fish lateral line is the nerve mound which is mainly distributed on the epidermis of a fish (referred to as the body surface nerve mound) and the base inside the duct (called the duct nerve mound). The former mainly senses the speed and direction of the water flow, and the latter mainly senses the pressure gradient [1]. Inspired by this natural function in fishes, artificial lateral line sensor systems have been developed and applied to AUVs. Wolf et al. [1] simulated an artificial neural hill and developed an all-optical 2D flow sensor to determine the velocity and direction of a fluid field. Chen et al. [2] developed an artificial ciliated cell sensor and found a detection limit of 1 mm/s of the sensor. Liu et al. [3] discussed the techniques of constructing artificial cilia sensors such as semiconductors, metals, and polymers. Fan et al. [4] completed the design and manufacture of a single fish lateral line sensor based on micromechanical distribution. Han et al. [5] classified artificial hair sensors into different working principles based on an animal hair sensor and fish lateral line. Izadi et al. [6] designed and fabricated flexible film arrays with a diameter of 100 μm, a gap height of 3 μm, and a mutual distance of 200 μm.

The accuracy when sensing the fluid environment using a single sensor is relatively low. An artificial lateral line system (ALLS) integrates a sensor array to provide much more reliable performance for underwater environment perception. Yang et al. [7] developed an ALLS using artificial cilia sensors and micro-electromechanical system technology to successfully locate artificial dipole sources. Fu et al. [8,9] and Jiang et al. [10] laminated polypropylene and polyvinylidene fluoride (PVDF) layers to form cantilever flow sensing elements which were used to develop the ALLSs. The positioning of the dipole source was achieved. Boulogne et al. [11] used neural networks to identify the location of underwater objects extracted from an ALLS. Ahmad et al. [12,13,14] proposed a new ion-exchange polymer metal composite (IPMC)-made sensor distributed in an array on a cylinder to sense underwater dipole sources. Abels et al. [15] used a linear array of pressure strained gage-based cantilever beams to simulate artificial neural lines to measure local fluid velocity, propagation velocity, and fluid direction. Equipped with these different sensor-based ALLSs, AUVs can achieve effective environment perception. Lin et al. [16] used fluid dynamics to study the spatial distribution of surface pressure of an AUV at different speeds. Salumae et al. [17] applied a piezoresistive pressure sensor-based ALLS to an AUV to estimate the surrounding fluid velocity. Fukuda et al. [18] tested four AUV models in classifying the watershed via an ALLS. Similarly, Chambers et al. [19] and Akanyeti et al. [20] estimated the surrounding fluid characteristics using an ALLS-AUV. Chen et al. [21] used a fish-shaped probe equipped with an artificial lateral line to measure a complex flow in turbulent environment. Yen et al. [22] controlled the movement of a fish robot with necessary flow information provided by an ALLS. Wang et al. [23] mounted an ALLS on the surface of a robotic fish to predict the robot velocity. Zheng et al. [24] equipped an ALLS to a fish robot to detect the reverse Karman street vortex wake produced by its adjacent robotic fish. Venturelli et al. [25] installed rigid pressure sensors with directional distribution on a rigid carrier, the pressure sensors can perceive the position and attitude of the carrier and calculate the hydrodynamics between the carrier and the flow field. Wissman et al. [26] developed a capacitive bio-inspired flow sensor and proposed an energy calculation relationship between the flow and capacitance. Because the flow sensor can comply with the movement of the carrier, their investigation suggested that the flow sensor is suitable for flexible robots.

Although progress has been made on the structure and function of artificial lateral lines, the development of ALLS is still in the early stage. For example, most of developed ALLSs are tested with static carriers. In order to achieve more realistic imitation of fish activities, an AUV-shaped carrier with an ALLS is proposed in this paper. The sensing ability of the moving carrier under different environmental conditions was investigated, and estimation of fluid velocity, carrier speed, and flow angle were performed. Numerical simulation and experimental evaluation were carried out to demonstrate the feasibility of the proposed ALLS-AUV.

## 2. Proposed ALLS-AUV Model

### 2.1. Model Establishment

**Figure 1:**
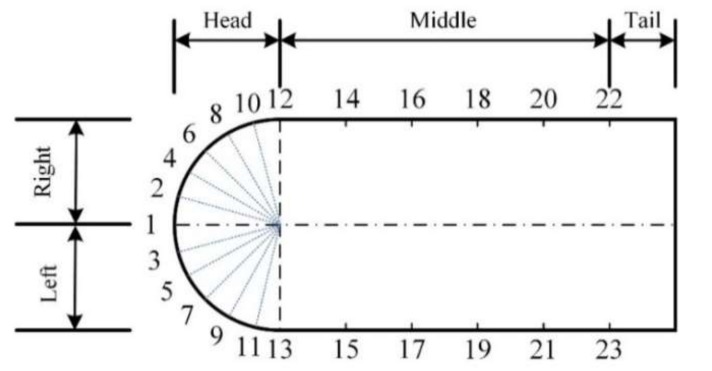
Sensor position distribution on the horizontal plane of the carrier.

The AUV-shaped carrier was designed with a length of 378 mm and a diameter of 160 mm as shown in Figure 1. Since the static and dynamic pressures were substantially the same in the cross-section perpendicular to the carrier axis [27], the carrier could be simplified as a two-dimensional model. The horizontal plane of the carrier is shown in Figure 1, and the sensors are distributed along the horizontal plane. Given the factor that the neuromasts of a fish are mainly distributed around the head area, the sensor density at the carrier head should be much larger than other areas. Considering the physical dimensions of the sensors, in this work, the head sensors were evenly distributed at an interval of 15°, and the arc length between adjacent sensors was approximately 25 mm. In the middle and the tail of the carrier, the sensors were evenly distributed at a pitch of 50 mm. Taking the axis in Figure 1 as a dividing line, the top part of the carrier is the right side of the carrier forward direction and the bottom part is the left side. As can be seen in Figure 1, the carrier is symmetrical to the dividing line.

**Figure 2:**
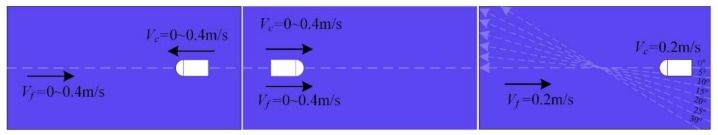
Schematic diagram of working conditions between the fluid velocity (*V_f_*) and carrier speed (*V_c_*).

The pressure law of the AUV-shaped carrier is studied in the horizontal direction. Considering the identical configuration of the carrier, a two-dimensional model of the carrier and the fluid can achieve comparable calculation performance to a three-dimensional model. Hence, in this study, a two-dimensional model was used to perform simulations under different flow angles between the fluid velocity direction and the carrier navigation direction as shown in Figure 2.

**Figure 3:**
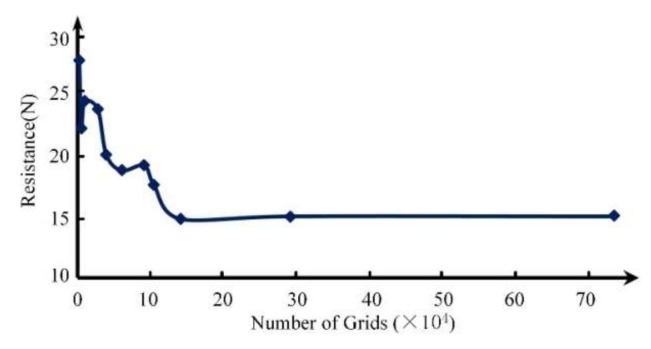
Grid independence verification.

**Figure 4:**
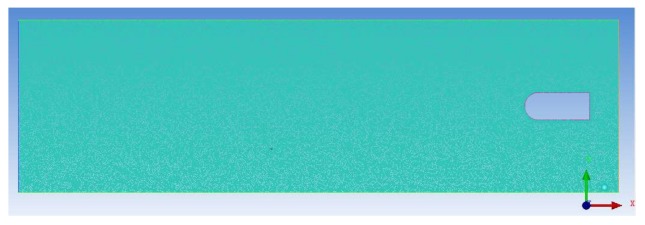
Carrier model and calculated fluid domain.

During the mesh division, because of the boundary layer on the surface of the carrier, it is necessary to carry out mesh encryption on the near-wall surface of the carrier. Grid independence was verified in this paper as shown in Figure 3. When the number of mesh was approximately 130,000, the resistance of the carrier started to remain stable. Therefore, the number of grids selected was 146,485 in this study. Figure 4 shows the carrier model and the fluid domain. Because the carrier was moving, dynamic mesh technology was required. In this study, the combined method of the spring smoothing and the re-meshing was used to update the model meshing. The basic parameters of the simulation are shown in Table 1.

In fluent simulation, the actual pressure on the measure point of the carrier was calculated, while an experimental prototype was tested in the same operating conditions to record the absolute pressure values (i.e., exclude the atmospheric pressure of 101,325 Pa). In order to compare the simulation and experimental measurements, one must make these pressure measurements consistent. For this reason, the operation pressure was set to 101,325 Pa in the fluent simulation, and the fluent static pressure could be calculated by subtracting the operation pressure from the actual pressure. By doing so, the simulation calculation was consistent with the experimental measurement

### 2.2. Simulations in Different Flow Fields

In an actual marine environment, the navigation environment of AUVs is very complex. The fluid velocity and wave are two significant factors that affect the flow field. In the simulation, the fluid velocity (*V_f_*), carrier speed (*V_c_*), and flow angle were analyzed. The flow angle is the angle between the direction of fluid velocity and the sailing direction of the carrier.

#### 2.2.1. Analysis of *V*_f_ and *V*_c_

For the analysis of *V_f_* and *V_c_*, the simulations were carried out in the same direction and the opposite direction of these two parameters. In both cases, the carrier sailed at different speeds of 0.1, 0.2, 0.3, and 0.4 m/s with different fluid velocities of 0.1, 0.2, 0.3, and 0.4 m/s, respectively.

(1) *V_f_* and *V_c_* in the opposite direction

When *V_f_* and *V_c_* are in the opposite direction, the working conditions of *V_f_* = 0.1 m/s and *V_c_* = 0.2 m/s, *V_f_* = 0.2 m/s and *V_c_* = 0.1 m/s, *V_f_* = 0.2 m/s, and *V_c_* = 0.2 m/s were analyzed. Moreover, the following two working conditions were also considered: the carrier is stationary in the fluid (where *V_f_* selected 0.3 m/s and 0.4 m/s, respectively, corresponding to the sum of *V_f_* and *V_c_* in the above working conditions) and the carrier sails in still water (where *V_c_* selected 0.3 m/s and 0.4 m/s, respectively, corresponding to the sum of *V_f_* and *V_c_* in the above working conditions).

**Figure 5:**
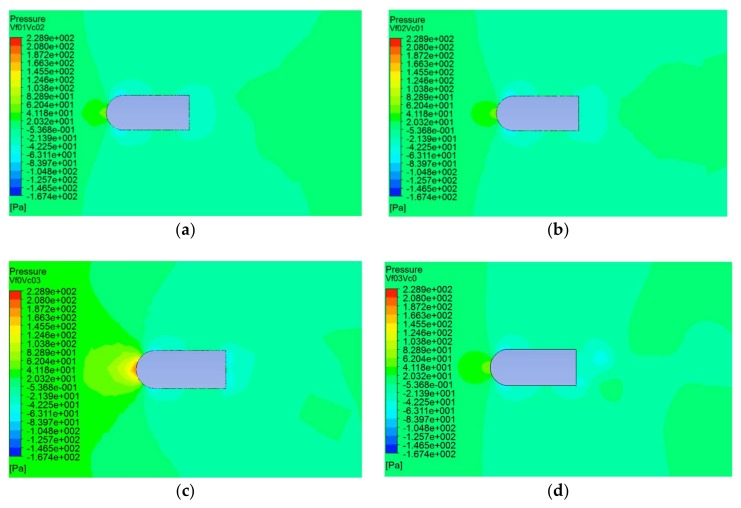

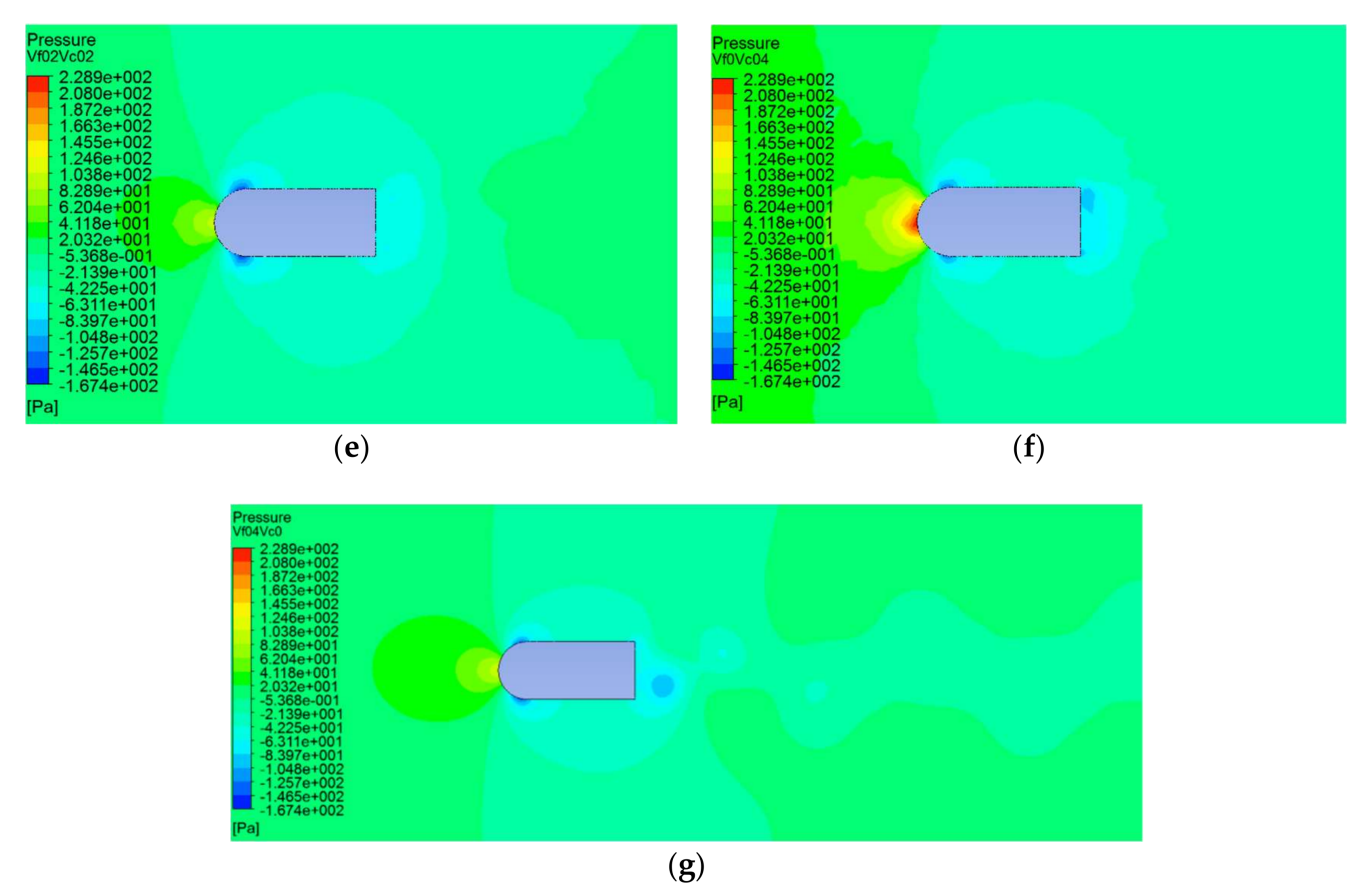
Static pressure distributions in different simulation conditions. (**a**) *V_f_* = 0.1 m/s and *V_c_* = 0.2 m/s; (**b**) *V_f_* = 0.2 m/s and *V_c_* = 0.1 m/s; (**c**) *V_f_* = 0 m/s and *V_c_* = 0.3 m/s; (**d**) *V_f_* = 0.3 m/s and *V_c_* = 0 m/s; (**e**) *V_f_* = 0.2 m/s and *V_c_* = 0.2 m/s; (**f**) *V_f_* = 0 m/s and *V_c_* = 0.4 m/s; (**g**) *V_f_* = 0.4 m/s and *V_c_* = 0 m/s.

Figure 5 shows the simulation results. As can be seen in Figure 5, the pressure distribution around the carrier was very similar in different working conditions. The maximum static pressure was at the head apex and the minimum value was at the intersection of the head and the middle parts of the carrier. However, In Figure 5a–d, the relative velocity of the carrier and the flow was 0.3 m/s, and, obviously, *V_c_* plays a leading role in Figure 5a,c, where the pressure value on the surface of the carrier is higher than other positions; *V_f_* plays a leading role in Figure 5b,d, where the turbulent vortex street at the tail of the carrier is more obvious. The observations are the same in Figure 5e,f,g. In addition, when the relative velocity of the carrier and water was 0.4 m/s, the static pressure was higher than that when the relative velocity was 0.3 m/s.

**Figure 6:**
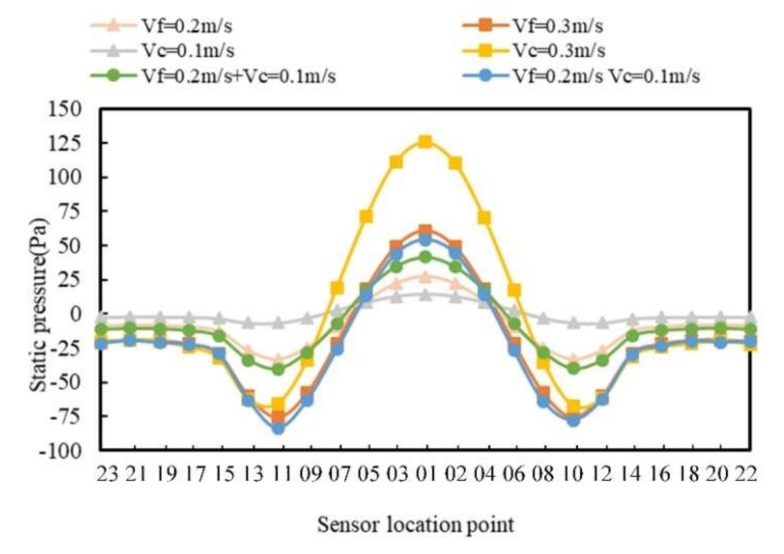
Static pressure distribution curve of the *V_f_* and *V_c_* in the opposite direction.

Affected by the fluid velocity and carrier speed, the pressure distribution around the carrier was further analyzed. The pressure distribution curve of *V_f_* = 0.2 m/s and *V_c_* = 0.1 m/s was drawn below, as well as the curves in other conditions, as shown in Figure 6.

**Figure 7:**
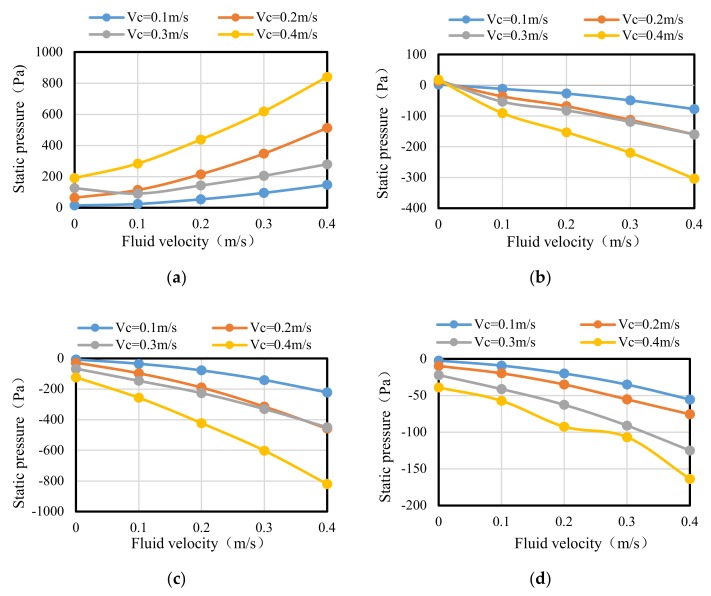
Static pressure curves at key points. (**a**) Sensor 1; (**b**) Sensor 6; (**c**) Sensor 10; (**d**) Sensor 18.

As can be seen from Figure 6, the curves of *V_f_* = 0.2 m/s and *V_c_* = 0 m/s and *V_f_* = 0 m/s and *V_c_* = 0.1 m/s are different from the curve of *V_f_* = 0.2 m/s and *V_c_* = 0.1 m/s. Therefore, this indicates that the pressure generated by the simultaneous movement of the water flow and the carrier were not the superposition of the pressure generated by the two single movements of the water flow and the carrier. In addition, it can be seen more clearly from the graph that, when the carrier speed was larger, the pressure on the surface of the carrier was higher. By comparing the curves in Figure 6, the pressure at sensor 1 was the largest, the pressure curve of each sensor was symmetric at the right and left sides, and the pressure at sensors 10 and 11 were the smallest. On the right side, the pressure at sensor 6 changed drastically. In order to analyze the change in pressure at specific positions with the increase of fluid velocity, key positions at sensors 1, 6, 10, and 18 were selected to draw the static pressure curves at different fluid velocities and different carrier speeds as shown in Figure 7.

It can be seen from Figure 7a that the static pressure at sensor 1 increased with the increase of fluid velocity, and the faster the carrier speed the greater the static pressure. As can be seen at sensor 6 in Figure 7b, sensor 10 in Figure 7c, and sensor 18 in Figure 7d, the static pressure decreased with the increase of fluid velocity, and with the increase of the carrier speed the static pressure dropped.

(2) *V_f_* and *V_c_* in the same direction

**Figure 8:**
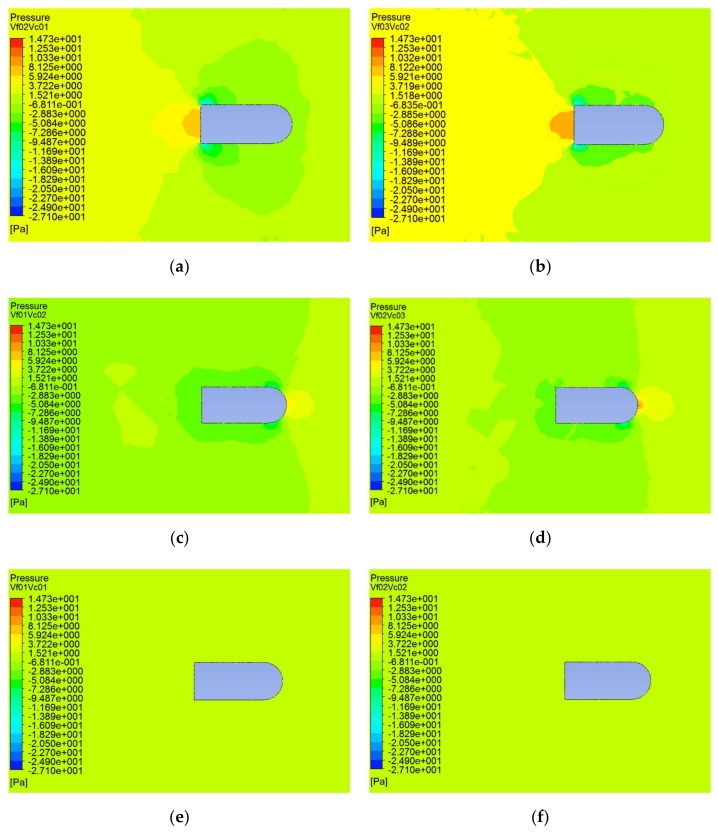
Cloud diagram of pressure distribution under different working conditions. (**a**) *V_f_* = 0.2 m/s and *V_c_* = 0.1 m/s; (**b**) *V_f_* = 0.3 m/s and *V_c_* = 0.2 m/s; (**c**) *V_f_* = 0.1 m/s and *V_c_* = 0.2 m/s; (**d**) *V_f_* = 0.2 m/s and *V_c_* = 0.3 m/s; (**e**) *V_f_* = 0.1 m/s and *V_c_* = 0.1 m/s; (**f**) Vf = 0.2 m/s and *V_c_* = 0.2 m/s.

When *V_f_* and *V_c_* were in the opposite direction, according to the relative relationship between the fluid velocity and the carrier speed, the simulation conditions can be divided into three groups: (1) the carrier speed was greater than the fluid velocity; (2) the carrier speed was less than the fluid velocity; and (3) the carrier speed was equal to the fluid velocity. Figure 8 shows the cloud diagram of the pressure distribution under these working conditions.

As can be seen from Figure 8a,b, when *V_f_* was greater than *V_c_*, the fluid velocity played a leading role, and the pressure on the carrier mainly distributed at the tail of the carrier. The pressure on two sides of the tail was the smallest, while the pressure was relatively large in the middle of the tail and was the largest at the tail end. In Figure 8c,d, when *V_f_* was smaller than *V_c_*, the carrier movement played the leading role, the pressure at the top of the head was the greatest, and at the junction between the two sides of the head and the middle body, the pressure was the smallest. In Figure 8e,f, when *V_f_* was equal to *V_c_*, the pressure in the flow field and on the surface of the carrier was uniformly distributed.

**Figure 9:**
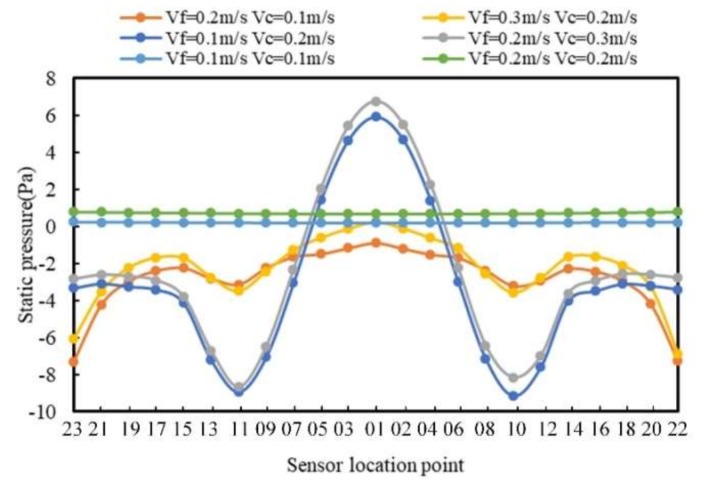
Static pressure distribution curve of the *V_f_* and *V_c_* in the same direction.

In order to further analyze the pressure distribution of the sensor position, the pressure curves of all the sensors are shown in Figure 9.

It can be seen from Figure 9 that the curves are divided into three types which are consistent with the classification patterns in the cloud map in Figure 8. When *V_f_* is greater than *V_c_*, the overall change in the pressure was volatile, but the fluctuation was small; the pressure of the sensor was the minimum on both sides of the tail and was the maximum on the top of the head. When *V_f_* was less than *V_c_*, the pressure curve changed significantly, and the pressure at the top of the head was the largest. The minimum static pressure was located on both sides of the head. The closer to the tail, the more stable the static pressure. When *V_f_* was equal to *V_c_*, the pressure of the sensor on the surface of the cylindrical carrier was constant and increased with the increase of the fluid velocity and the carrier speed.

(3) Discussions

Theoretically, when an object performs uniform linear motion relative to fluid, its surrounding pressure field is basically determined by its relative speed to the fluid in a steady flow [28,29]. This phenomenon can be described using Bernoulli’s principle:

(1) $$\frac{P_{1}}{\rho }+z_{1}g+\frac{v_{1}{}^{2}}{2}=\frac{P_{2}}{\rho }+z_{2}g+\frac{v_{2}{}^{2}}{2}$$

where, *v*_1_ and *v*_2_ are respectively the flow speed and the carrier speed; *P*_1_ and *P*_2_ are respectively the flow pressure and the carrier pressure; *z*_1_ and *z*_2_ are respectively the elevation of the flow and the carrier; *g* is the gravitational acceleration and *ρ* is the fluid density. It should note that Equation (1) ignores the friction by viscous forces. Under this circumstance, the obtained pressure curves should be very similar because the relative speed between the flow and carrier is the same for the three setting-ups. However, in this study the turbulent model was used in the simulation, and hence, the flow was in unsteady condition. In this circumstance, the Bernoulli’s principle should be described as

(2) $$\frac{P_{1}}{\rho }+z_{1}g+\frac{\alpha_{1}v_{1}{}^{2}}{2}=\frac{P_{2}}{\rho }+z_{2}g+\frac{\alpha_{2}v_{2}{}^{2}}{2}+h_{w}g$$

where *α*_1_ and *α*_2_ are the kinetic energy correction coefficients of the flow and the carrier, and *h_w_* is the energy loss caused by viscosity. In Equation (2), the pressure is affected by the velocity potential with respect to time and the energy loss. One can note in Figure 1 that the head of the carrier is an arc shape which will generate a non-linear change in the velocity potential. As a result, even though the relative speed between the flow and carrier is the same, the calculated pressure may vary according to the specific situation. Moreover, because of the Reynolds-averaged Navier–Stokes equations (RANS) form of the RNG *k–ε* turbulent model in the simulation, the relationship between the pressure, flow, and carrier velocities is non-linear according to the RANS equations. Therefore, although the relative velocity values are the same, the calculated pressure is not simply generated by the sum of the flow and carrier velocities. As a result, it can be seen in Figure 5, Figure 6, Figure 7, Figure 8 and Figure 9 that even if the relative velocity between the carrier and the flow is equal, the pressure distribution on the surface of the carrier is slightly different.

#### 2.2.2. Analysis of flow angle

**Figure 10:**
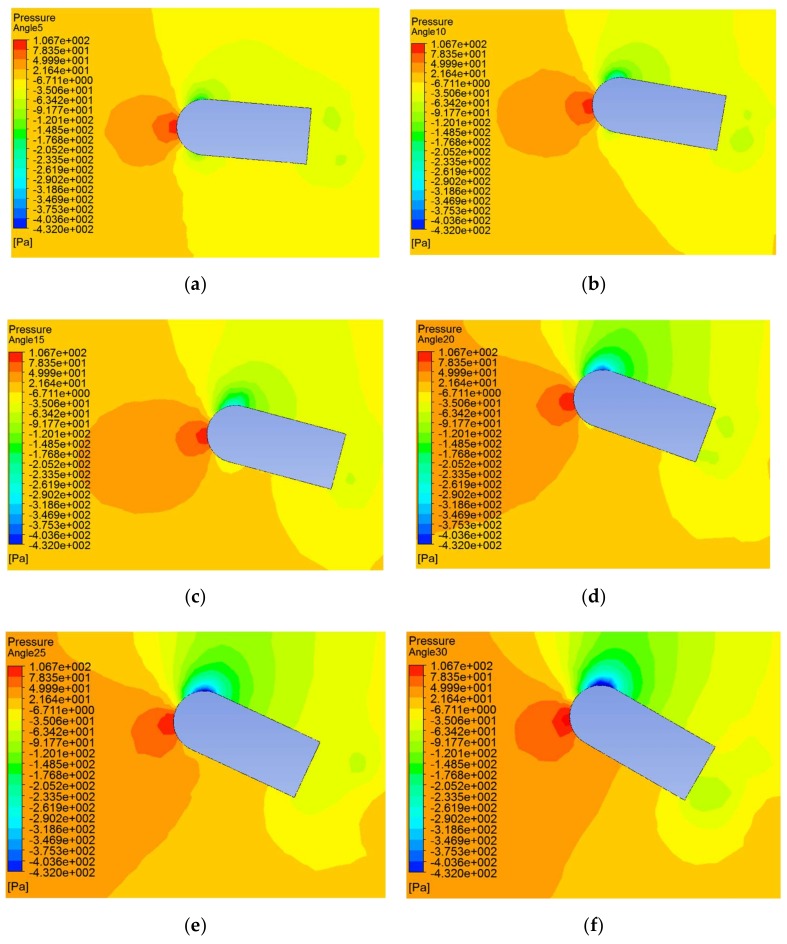
Static pressure distributions at different flow angles. (**a**) A flow angle of 5°; (**b**) A flow angle of 10°; (**c**) Flow angle is 15°; (**d**) Flow angle is 20°; (**e**) A flow angle of 25°; (**f**) A flow angle of 30°.

Furthermore, the pressure distributions were obtained with different flow angles of 5°, 10°, 15°, 20°, 25°, and 30°. Figure 10 shows the pressure distribution clouds of the flow field at these angles at the carrier speed of 0.2 m/s and the fluid velocity of 0.2 m/s.

In Figure 10, the direction of fluid velocity is from the left to the right side of the carrier. As can be seen from Figure 10, no matter the flow angle, the maximum static pressure was still at the incident flow surface of the carrier, and the area of the maximum pressure expanded with the increase of the flow angle. The position of the minimum pressure changed with the flow angle. When the flow angle was small, the position of the minimum static pressure was at the intersection of the head and the middle. As the angle increased, the area of the minimum static pressure on the left side of the carrier gradually shrunk until it disappeared as shown in Figure 10c. However, the position of the minimum static pressure on the right side remained with the flow angle.

**Figure 11:**
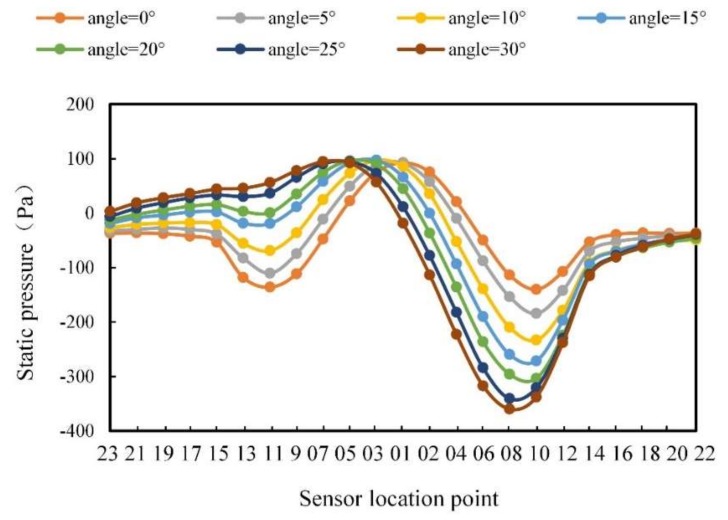
Static pressure curve for different flow angles.

In order to specifically describe the change in the pressure on the carrier surface with the change of angle, the static pressure curves of 23 sensor points are drawn in Figure 11.

It can be seen from Figure 11 that when the angle was 0°, the static pressure change trend was symmetrically distributed on the two sides of the carrier. The maximum static pressure was located at point 1, while the minimum static pressure was located at points 10 and 11. By contrast, the static pressure presented a steady state in the second half of the middle part (such as points 16, 18, 20, and 22). As the angle increased, the maximum static pressure gradually moved to the left side of the carrier, while the minimum value on the left side remained; the static pressure gradually increased until the minimum value disappeared; the position of the right minimum value moved slightly to the left, and the static pressure gradually decreased.

## 3. Experimental Evaluation

### 3.1. Experiment Process

**Figure 12:**
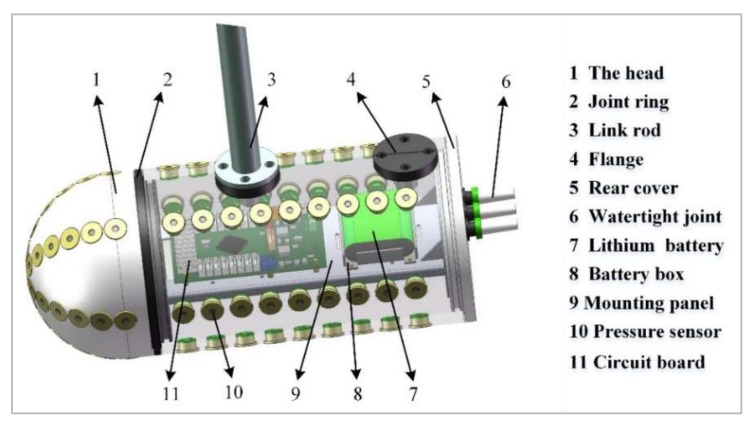
3D picture of the carrier.

**Figure 13:**
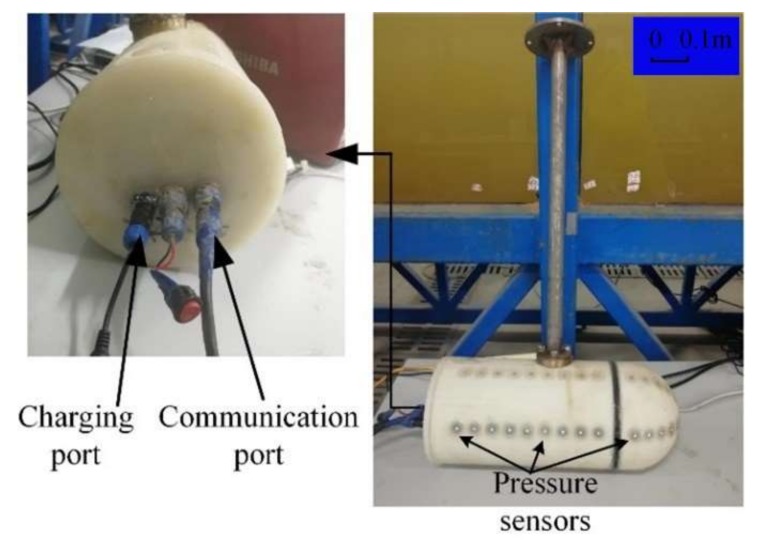
Images of the carrier.

In order to verify the simulation model, an experimental model was manufactured. The 3D drawing and images of the experimental model are given in Figure 12 and Figure 13, respectively. The ALLS consisting of a pressure sensor array was mounted on a horizontal surface of the carrier over the axis. The pressure sensor used in this system was MS5803-07BA. The resolution of the sensor was 0.04 mba, and the analog-to-digital (DA) conversion response time was 8.2 ms. Due to the limitation of the DA conversion time of the digital sensor, the signal sampling frequency of the ALLS was 9.524 HZ. The STM32F103ZET6 master control chip was arranged inside the carrier to collect the pressure data. Specific parameters of the carrier are shown in Table 2.

Prior to the test, a standard measurement of the pressure sensor was required. The difference between the pressure of the sensor in the atmosphere and the pressure at a certain water depth *h* was measured, and then the measurement was compared with the theoretical calculation of the static pressure. The comparison showed that the two results were basically the same, indicating the validness of the sensor data.

**Figure 14:**
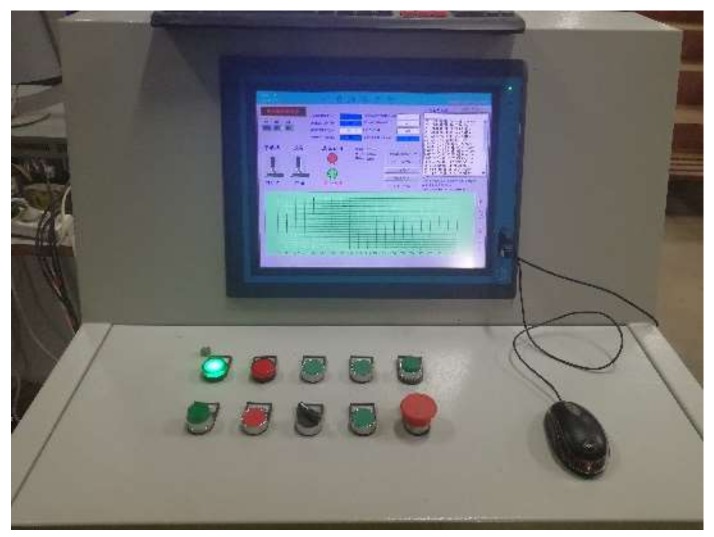
Flow control system.

The experiment tests were carried out in a flume at the Engineering Hydrodynamics Laboratory, Ocean University of China. The flume was 30 m in length, 1 m in width, and 1 m in depth. The maximum fluid velocity was 0.8 m/s. The water in the flume was fresh water. The flow control system is shown in Figure 14.

**Figure 15:**
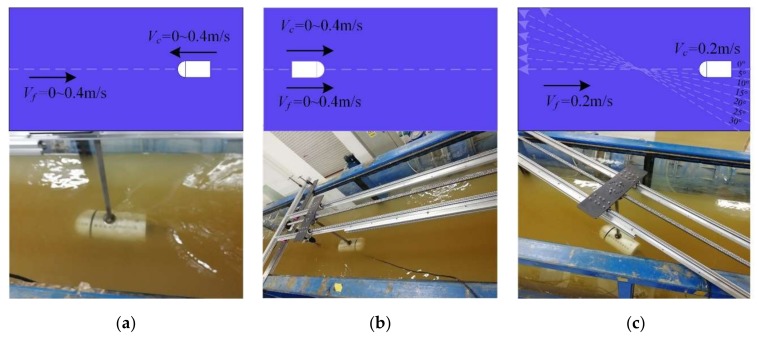
Experimental process. (**a**) Opposite direction; (**b**) Same direction; (**c**) Different angles.

The working conditions of the experiment tests were consistent with the simulation conditions, where (a) *V_c_* and *V_f_* were in the opposite direction; (b) *V_c_* and *V_f_* were in the same direction; and (c) different flow angles between *V_c_* and *V_f_* were at a constant fluid velocity of 0.2 m/s and constant carrier speed of 0.2 m/s. The experiment process is shown in Figure 15.

### 3.2. Experimental Results

The pressure data under the same working conditions as in the simulation were obtained through the flume experiments. The testing process is always influenced by the environment, equipment, and sensor noise. Therefore, data pre-processing is required to reduce the influence of these interference factors. One way is to average the experimental data to reduce the influence of instantaneous pressure fluctuation and remove the hydrostatic pressure.

#### 3.2.1. V_f_ and V_c_ in the Opposite Direction

**Figure 16:**
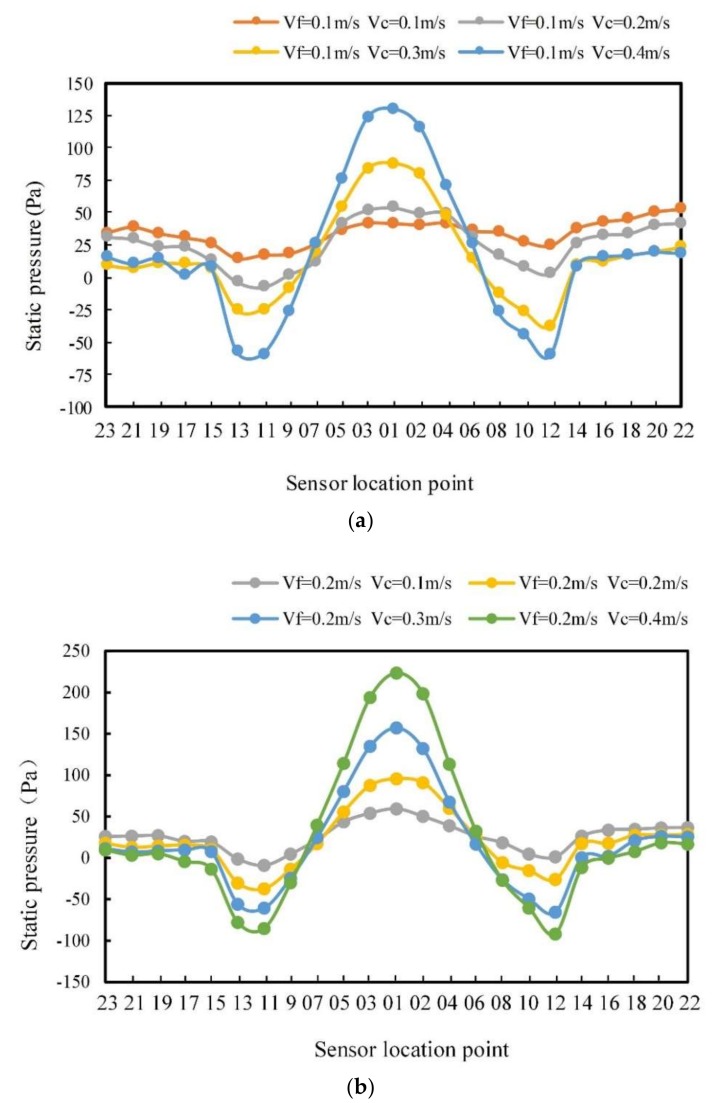
Sensor pressure distribution at different carrier speeds: (**a**) fluid velocity is 0.1 m/s, and (**b**) fluid velocity is 0.2 m/s.

The pressure data were obtained under the same conditions as in the simulation. Figure 16 shows the pressure distributions with the carrier speeds of 0.1–0.4 m/s and the fluid velocities of 0.1 and 0.2 m/s.

As can be seen in Figure 16a, the carrier sailed at a speed of 0.1 to 0.4 m/s in a fluid of 0.1 m/s. The pressure at point 1 was the largest. As the carrier speed increased, the pressure increased. The minimum pressure appeared at points 11 and 12, and the pressure decreased as the carrier speed increased. The pressure changing from point 18 to point 22 on the right side was gentle. On the other hand, as the speed of the carrier increased, the extreme value of the pressure increased, and the change was more severe. However, it was found that there were very small differences in the shape of pressure curves between the experiment and the simulation. This may be due to the atmospheric pressure instability, water quality, and other environmental factors during the experimental tests.

Figure 16b shows the pressure curve of the carrier at the fluid velocity of 0.2 m/s. Compared with Figure 16a, the maximum pressure increased and the minimum pressure decreased with the increase in the fluid velocity. The pressure variation range became larger when the fluid velocity increased from 0.1 to 0.2 m/s. Therefore, the pressure on the surface of the carrier increased as the fluid velocity increased. Comparing Figure 10 with Figure 3, one can note that the experimental conclusion results are consistent with the simulation results which verifies the correctness of the simulation model.

#### 3.2.2. V_f_ and V_c_ in the Same Direction

**Figure 17:**
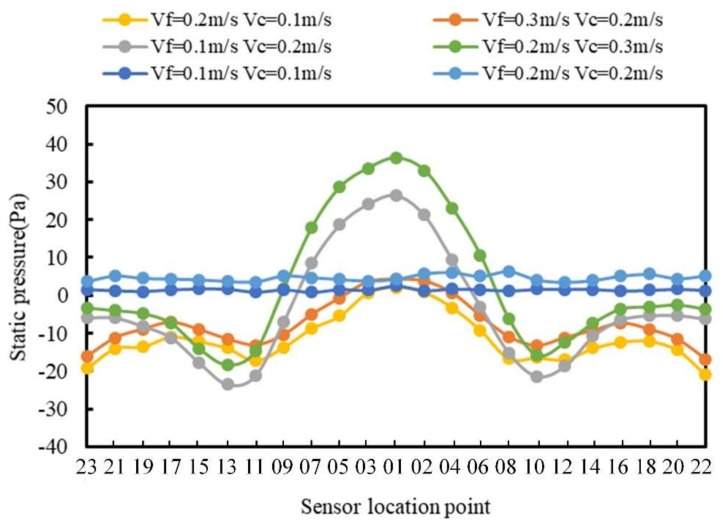
Pressure distribution when *V_f_* and *V_c_* are in the same direction.

The obtained pressure data were for when *V_f_* and *V_c_* were in the same direction are plotted and analyzed in Figure 17.

In Figure 17, the pressure distribution includes three conditions, i.e., *V_f_* is less than, greater than, and equal to *V_c_*. When *V_f_* was less than *V_c_*, the movement of the carrier played a major role. The pressure change trend was similar to the change trend when the fluid direction and the carrier direction were in the opposite direction. When *V_f_* was greater than *V_c_*, the pressure change range became small; however, the change in the tail became significant. Closer to the tail, the smaller the pressure. When *V_f_* was equal to *V_c_*, the pressure basically remained unchanged, the distribution was relatively uniform, and the pressure value only increased with the increase of the carrier velocity.

#### 3.2.3. Different Angles

**Figure 18:**
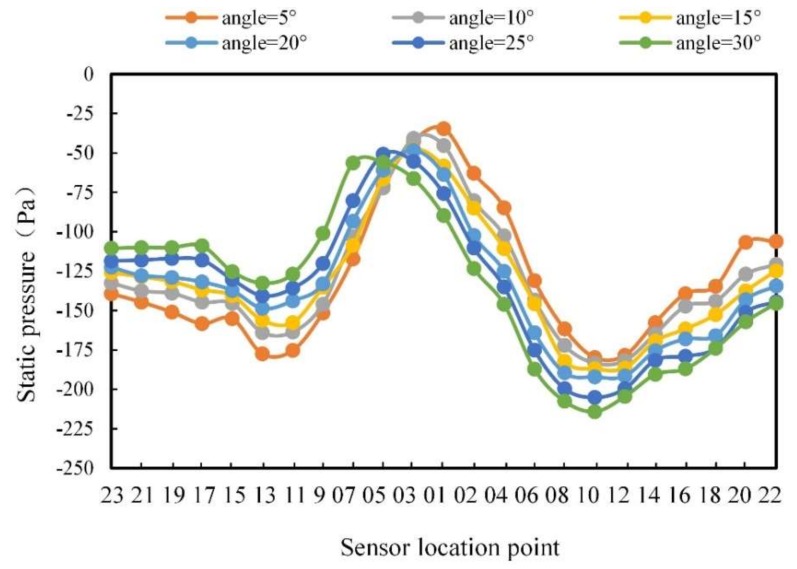
Pressure distribution at different flow angles.

Moreover, the pressure data were analyzed at different flow angles. Figure 18 shows the pressure curves with the flow angles of 5°, 10°, 15°, 20°, 25°, and 30°, respectively. The pressure distribution curves of the carrier surface in Figure 18 demonstrate different rules with the flow angle changing. The maximum pressure moved to the left side as the angle increased. The minimum pressure on the right side of the carrier gradually decreased as the angle increased. The minimum pressure on the left side of the carrier gradually increased as the angle increased. The pressures on both sides of the tail showed an asymmetrical change with the angle changing. The pressure on the left side of the tail changed gently with the increase of the flow angle. Comparing Figure 18 with Figure 11, it can be seen that the pressure change rule of the experimental results were basically consistent with that in the simulation, though there was a slight difference in value. The reason was probably that the atmospheric pressure in the laboratory was not stable during the experiments, and the turbulence and water quality also had an impact.

## 4. Flow Field Estimation

The pressure distribution diagram can be used to qualitatively determine the magnitude of fluid velocity, flow angle, and carrier speed, but quantitative values cannot be obtained. Three methods are introduced to predict the fluid field.

### 4.1. Fitting Method to Estimate Fluid Velocity

According to Figure 4, it can be seen that the static pressure was functionally related with the fluid velocity and the carrier speed. The carrier speed is known when the AUV sails. Therefore, the static pressure is only related with the fluid velocity which can be determined by a fitting method. According to the variation trend of the curve in Figure 4, it can be seen that the curve conforms to the Fourier function relation. Hence, the following relationship between the pressure and fluid velocity is proposed.

*P*_1_ = *a*_0_ + *a*_1_cos(*V_f_* * *w*) + *a*_2_sin(*V_f_* * *w*) (3)

where *P*_1_ is the static pressure, $a_{0}$, $a_{1}$, and $a_{2}$ are fitting coefficients, and *w* is a phase coefficient.

Taking points 1, 6, 10, and 18 as examples, the carrier speed was 0.2 m/s. The static pressure and fluid velocity data were fitted by MATLAB software. The obtained fitting coefficients are shown in Table 3.

**Figure 19:**
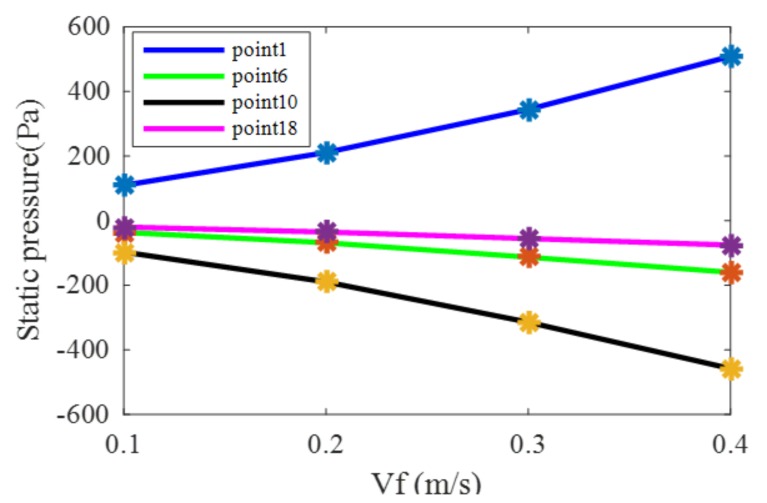
Fitting models.

The fitted models between the static pressure and fluid velocity at points 1, 6, 10, and 18 are shown in Figure 19. It can be seen from Table 2 that the *R*-squared determination coefficient is above 0.99, so the fitting models are acceptable for predicting the fluid velocity.

### 4.2. Differential Pressure Method to Estimate the Flow Angle

**Figure 20:**
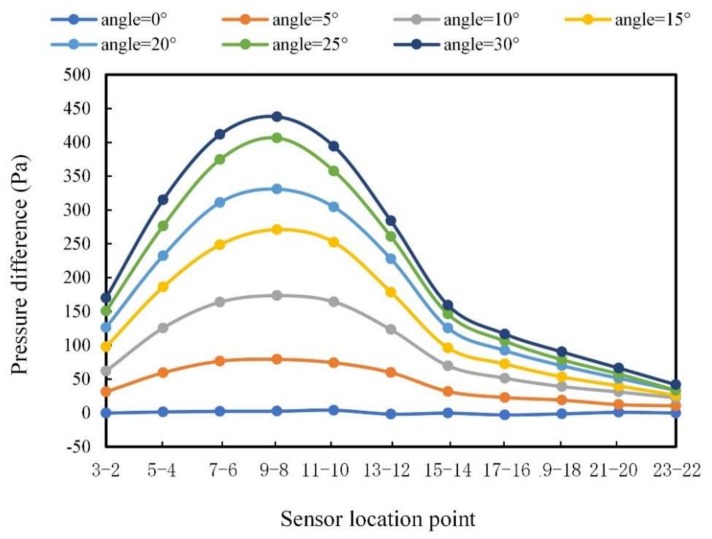
Pressure difference distributions.

When there is a certain angle between the carrier and the flow direction, the pressure difference between the left and right sides of the carrier can be used to predict the flow angle. Figure 20 shows the pressure difference of two symmetrical sensors on the left and right sides of the carrier with different flow angles. For example, symbol “3–2” indicates the pressure difference between point 3 and point 2.

**Figure 21:**
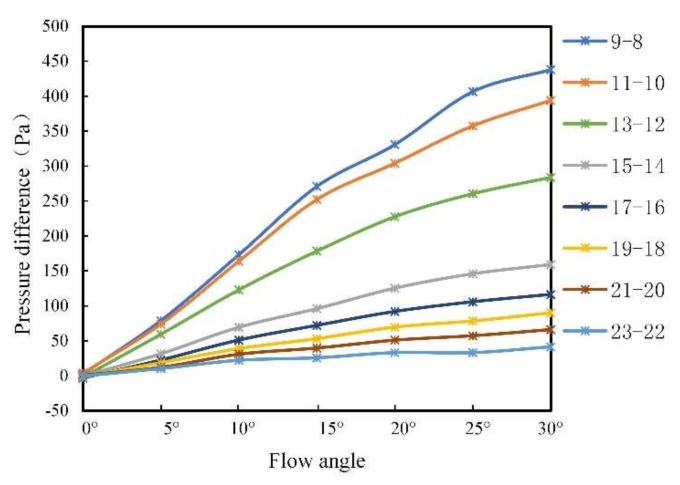
Pressure difference curves after truncation.

It can be seen from Figure 20 that the pressure difference distributions with different flow angles present a certain common change rule. Due to the symmetrical arrangement of the sensors on the left and right sides, there was no pressure difference with the flow angle of 0° (i.e., the pressure difference is 0 Pa). As the flow angle increased, the pressure difference increased. The position with the largest pressure difference locates at “9–8”. The closer to the top of the head the smaller the pressure difference. When close to the tail of the carrier, the pressure difference was small; that is, the influence of the flow angle was small. Furthermore, the pressure differences from “3–2” to “7–6” are symmetric to that from “15–14” to “11–10”. Based on this symmetric pattern, it is reasonable to only analyze one side of the pressure difference curves. In this work, the pressure difference data from point “9–8” to “23–22” were selected for analysis as shown in Figure 21.

As can be seen from Figure 21, an obvious pressure difference can be observed from “9–8” to “15–14”. Hence, these pressure difference data were used to establish the relationship between the pressure difference and flow angle. The relationship between the pressure difference *∆P* and the flow angle α is described as:

(4) $$\Delta P=p_{1}*\alpha^{2}+p_{2}*\alpha +p_{3}$$

where *p*_1_, *p*_2_, and *p*_3_ are quadratic parameters. Table 4 gives the obtained parameters.

Because the fitting coefficient *R*-squared in Equation (2) is close to 1, the prediction model can be used to predict the flow angle.

### 4.3. Estimating Flow Field with a Back Propagation (BP) Neural Network

**Figure 22:**
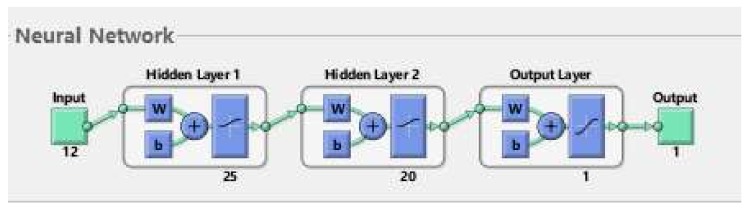
A back propagation (BP) neural network model.

In an actual marine environment, we sometimes cannot know the fluid velocity or the speed of AUVs in advance, but the ALLS can collect a series of data through pressure sensors. Since the pressure data rule formed under each working condition is different, a machine learning method can be sought to learn the complex data so as to predict the required parameters. In this paper, a BP neural network [30] was used to predict the fluid velocity, flow angle, and carrier speed. The structure of the BP neural network was 12 × 25 × 20 × 1, as shown in Figure 22. In the training process, because of symmetry, the 12 sensors on the right side of the carrier were used as the inputs of the recognition model. The output was the AUV working condition.

**Figure 23:**
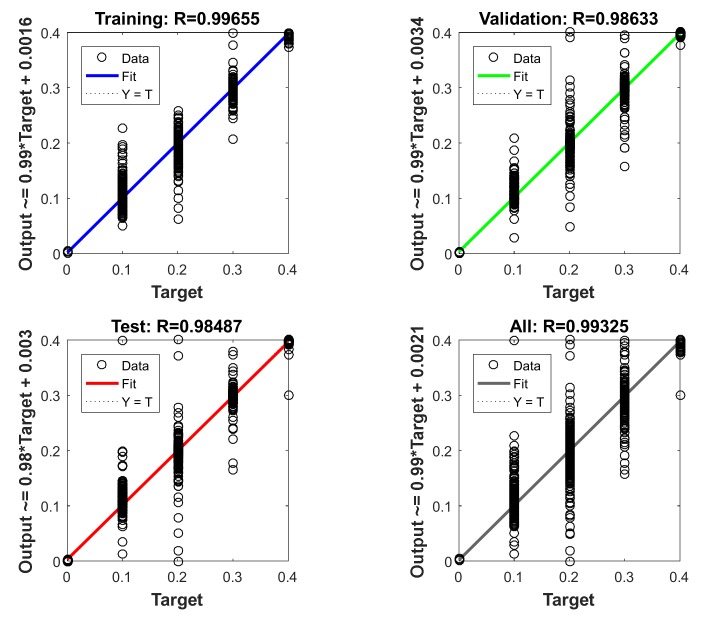
Fitting degree of expected results and test results of fluid velocity.

Firstly, when the *V_f_* and *V_c_* were in the opposite direction, the training models with fluid velocities of 0 m/s, 0.1 m/s, 0.2 m/s, 0.3 m/s, and 0.4 m/s were analyzed. Figure 23 shows that the fits of the training set, validation set, test set, and all set were up to 0.98, indicating that the model can be used to estimate the fluid velocity in an unknown flow field. Then, the data of the carrier speeds of 0 m/s, 0.1 m/s, 0.2 m/s, 0.3 m/s, and 0.4 m/s were trained. Similarly, the conditions of *V_f_* and *V_c_* in the same direction were also analyzed. Lastly, the data of the flow angles of 0°, 5°, 10°, 15°, 20°, 25°, and 30° were trained. Both models have a fitting accuracy of more than 0.98.

**Figure 24:**
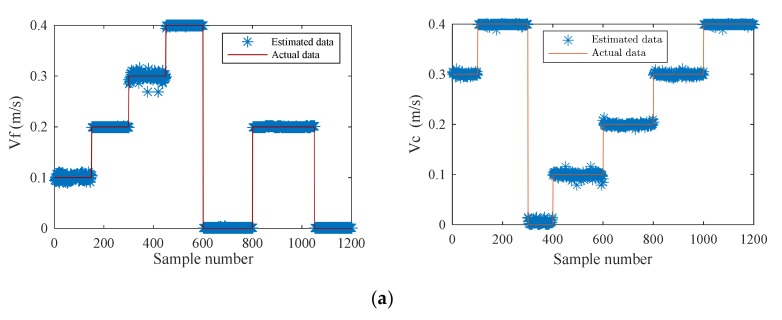

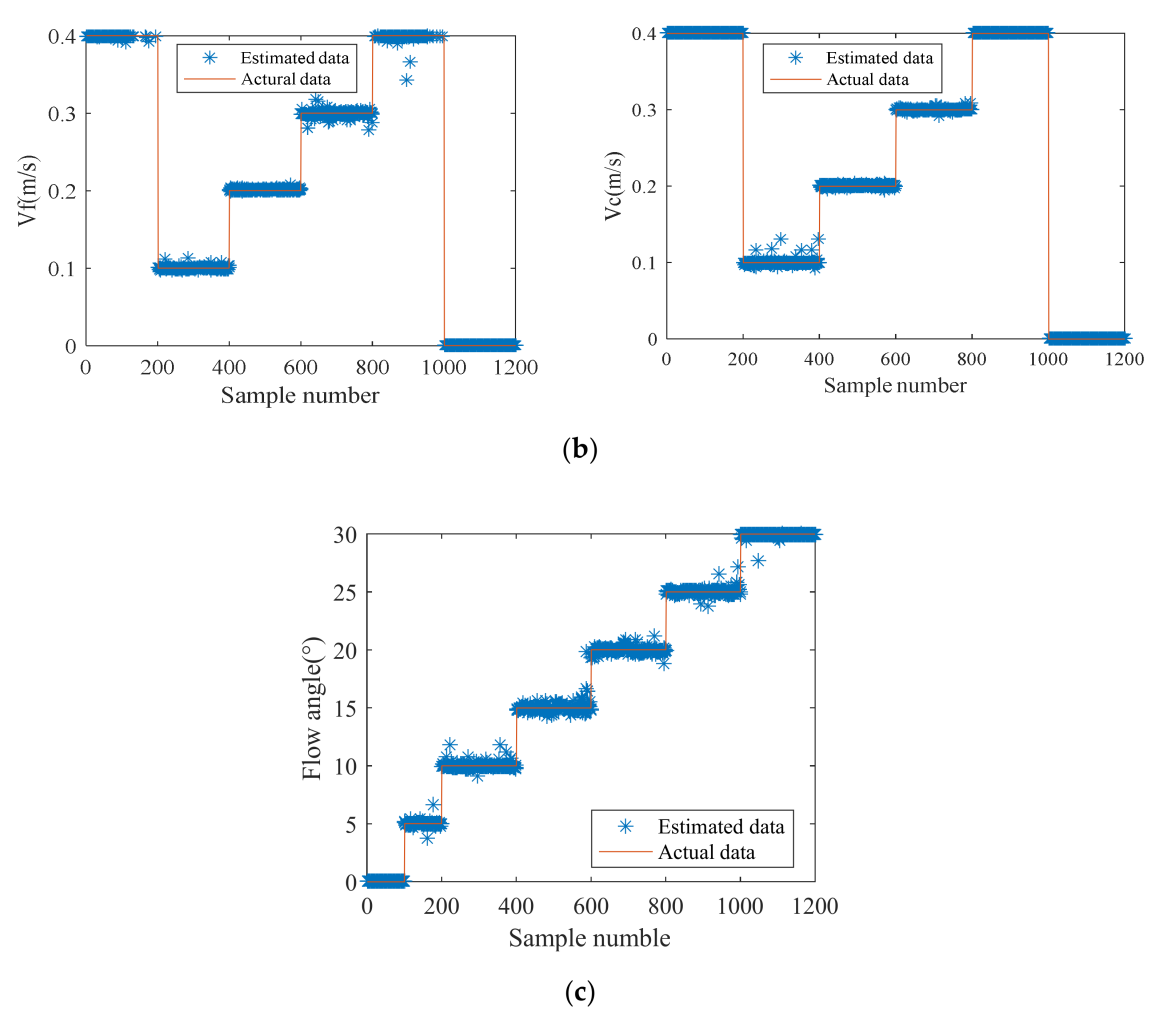
Comparison between estimated and actual results. (**a**) *V_f_* and *V_c_* in the opposite direction; (**b**) *V_f_* and *V_c_* in the same direction; (**c**) Flow angle.

In order to verify the effectiveness of the neural network model, 1200 new samples were used to estimate the fluid velocity; another 1200 new samples were used to estimate the carrier speed, and 1000 new samples were used to estimate the flow angle. The comparison between the estimated results and the actual results are shown in the Figure 24.

As can be seen from the Figure 24, the estimated data were distributed in the vicinity of the actual data. Only a few data deviated from the actual value, indicating that the overall recognition ability of the neural network model was relatively high. It can accurately predict the fluid velocity, carrier speed and flow angle.

By comparing the aforementioned three estimation methods, it can be seen that the fitting method and the differential pressure method are suitable for estimating the fluid velocity and the flow angle, while the BP model can simultaneously estimate the fluid velocity, the flow angle, and the carrier speed. As a result, when the fluid environment is unknown, it is more beneficial to use a BP neural network to predict the AUV working condition.

## 5. Conclusions

In this paper, a new ALLS-AUV was developed to recognize the fluid environment of a moving carrier. Both simulation and experimental tests were conducted to evaluate the pressure distributions of the ALLS. The analysis results demonstrate that, when *V_f_* and *V_c_* were in the opposite direction, the maximum pressure was on the carrier head; when *V_c_* was larger, the pressure value on the surface of the carrier was higher; when *V_f_* was larger, the turbulent vortex street at the tail of the carrier was more obvious; when the flow angle changed, the positions of the maximum pressure and minimum pressure varied. In addition, when the carrier speed was constant, a fitting model was proposed to predict the fluid velocity and a differential pressure method was introduced to predict the flow angle. In the unknown fluid environment, a BP neural network model was established to predict the fluid velocity, the flow angle, and the carrier speed. The recognition accuracy was over 99%. The findings of this study may provide the foundation for local perception, positioning, and navigation of AUVs in the ocean.

## Figures and Tables

**Table 1:** Basic parameters of the simulation.

**Mesh**			
**Fluid Dimensions**	**Global Element Scale Factor**	**Global Element Seed Size**	**Other Grid Sizes**
3 m *×* 1 m	1	100 mm	15 mm
**Carrier Boundary**			
The maximum size	Height of the first layer	Height ratio	The number of boundary layer
0.5 mm	0.01 mm	1.2	8
**Spring Smoothing**			
Spring constant factor	Convergence tolerance	Number of iterations	Elements
0.8	0.001	20	tri in tri zones
**Re-meshing**			
Maximum cell skewness	Maximum/minimum length scale	Sizing function	Resolution, variation, rate
0.7	default values	used in local grids	default values
**Computational Fluid Domain Material**			
Type	Material Name	Density	Viscosity
Incompressible Fluid	Water (liquid)	998.2 kg/m^3^	0.001003 kg/m∙s
**Hydrodynamic Simulation**			
Physical model	Boundary conditions	Reynolds number	Separation algorithm
Renormalization Group (RNG) *k-ε*	Velocity inlet/pressure outlet	>38,000	PISO (Pressure Implicit Split Operator)

**Table 2:** Specific parameters of the carrier.

**Project**	**Parameters**
Carrier size	*D* = 160 mm, *L* = 378 mm
Carrier material	Nylon and photosensitive resin
Sensor type	MS5803-07BA
Main control chip	STM32F103ZET6
Power supply module	3 × 3.7 V lithium battery
Switch	Watertight switch

**Table 3:** Fitting coefficients.

Points	1	6	10	18
*a*_0_	1.105 × 10^4^	−122.4	−496.9	−54.41
*a*_1_	−1.101 × 10^4^	98.99	450.8	41.39
*a*_2_	989	−2.714	−85.62	−2.641
*w*	0.5408	4.823	3.245	5.093
*R*-squared	1	0.9999	1	1

**Table 4:** Pressure difference and flow angle coefficient and parameters.

Points	9–8	11–10	13–12	15–14
*p*_1_	−0.188	−0.202	−0.1694	−0.08355
*p*_2_	20.77	19.47	14.82	7.959
*p*_3_	−7.428	−4.877	−5.635	−2.542
*R*-squared	0.9961	0.9968	0.9989	0.9984
